# Double Trouble: EPCAM Exon 9 Deletion in Lynch Syndrome Leading to Dual Primary Tumors

**DOI:** 10.7759/cureus.105528

**Published:** 2026-03-19

**Authors:** Areti Kalfoutzou, Cleopatra Rapti, Zannis Almpanis, Eleftheria Bagiokou, Nikolaos Kalakos, Vasiliki Lagopoulou, Vasileios Kolintzikis, Aristeidis Oikonomakis, Nikolaos Chaleplidis, Vasileios Ramfidis

**Affiliations:** 1 Department of Medical Oncology, 251 Air Force General Hospital, Athens, GRC; 2 Department of Pathology, Pathlabs - Pathology Laboratory of Athens, Athens, GRC; 3 Department of Gastroenterology, General Hospital of Athens "G. Gennimatas", Athens, GRC; 4 Department of Pathology, 251 Air Force General Hospital, Athens, GRC; 5 Second Department of Medical Oncology, Agios Savvas Cancer Hospital, Athens, GRC; 6 Department of Gastroenterology, 251 Air Force General Hospital, Athens, GRC

**Keywords:** dna mismatch repair, gene deletion, immunotherapy, lynch syndrome, msh2 protein

## Abstract

Lynch syndrome (LS) is one of the most widely recognized cancer susceptibility syndromes and is caused by germline mutations of the mismatch repair (MMR) genes *MLH1*, *MSH2*, *PMS2*, and *MSH6*. One of the less commonly known mechanisms is the deletion of the 3’ end of the Epithelial Cell Adhesion Molecule (*EPCAM*) gene, which is closely situated to the *MSH2* gene promoter. Such deletion causes hypermethylation of the* MSH2* gene promoter and leads to its inactivation. This case reports a young adult diagnosed with two metachronous primary tumors due to a germline *EPCAM* exon 9 deletion and subsequent *MSH2* gene inactivation, shedding light on one of the most underrecognised pathways of microsatellite instability.

## Introduction

Lynch syndrome (LS), also known as hereditary nonpolyposis colorectal cancer (HNPCC), is a hereditary cancer syndrome, accounting for about 3% of colorectal cancer (CRC) cases [1]. It is inherited in an autosomal dominant fashion, and is associated with a high cumulative risk for colorectal as well as extracolonic cancers, including endometrial, gastric, pancreatobiliary, urothelial, and skin cancers [2,3].

Apart from the known mismatch repair (MMR) genes, 3’ deletions of the Epithelial Cell Adhesion Molecule (*EPCAM*) gene are known to cause LS, by epigenetic silencing of the neighboring gene *MSH2* [3]. *EPCAM* gene alterations account for about 1-3% of diagnosed LS cases [3].

We report a case of a young adult male patient presenting with an early-stage, microsatellite instability (MSI)-high CRC, as well as a metastatic, microsatellite stable (MSS) pancreatobiliary adenocarcinoma more than a decade later, demonstrating an excellent response to chemo-immunotherapy. This case aims to highlight the *EPCAM* 3’ deletion as an underrecognized cause of LS, emphasizing the importance of comprehensive genetic testing for optimal diagnostic assessment.

## Case presentation

A 20-year-old male patient was referred to our institution for evaluation of blood in the stool for the past month. Clinical examination was negative for cafe-au-lait spots, axillary freckling, or pilomatrixomas. Lower gastrointestinal (GI) endoscopy with biopsies revealed a single exophytic polypoid lesion measuring 35 mm at the level of the hepatic flexure, indicative of a colon adenocarcinoma. Staging computed tomography (CT) scans of the chest and abdomen were negative for signs of metastatic disease.

**Figure 1:**
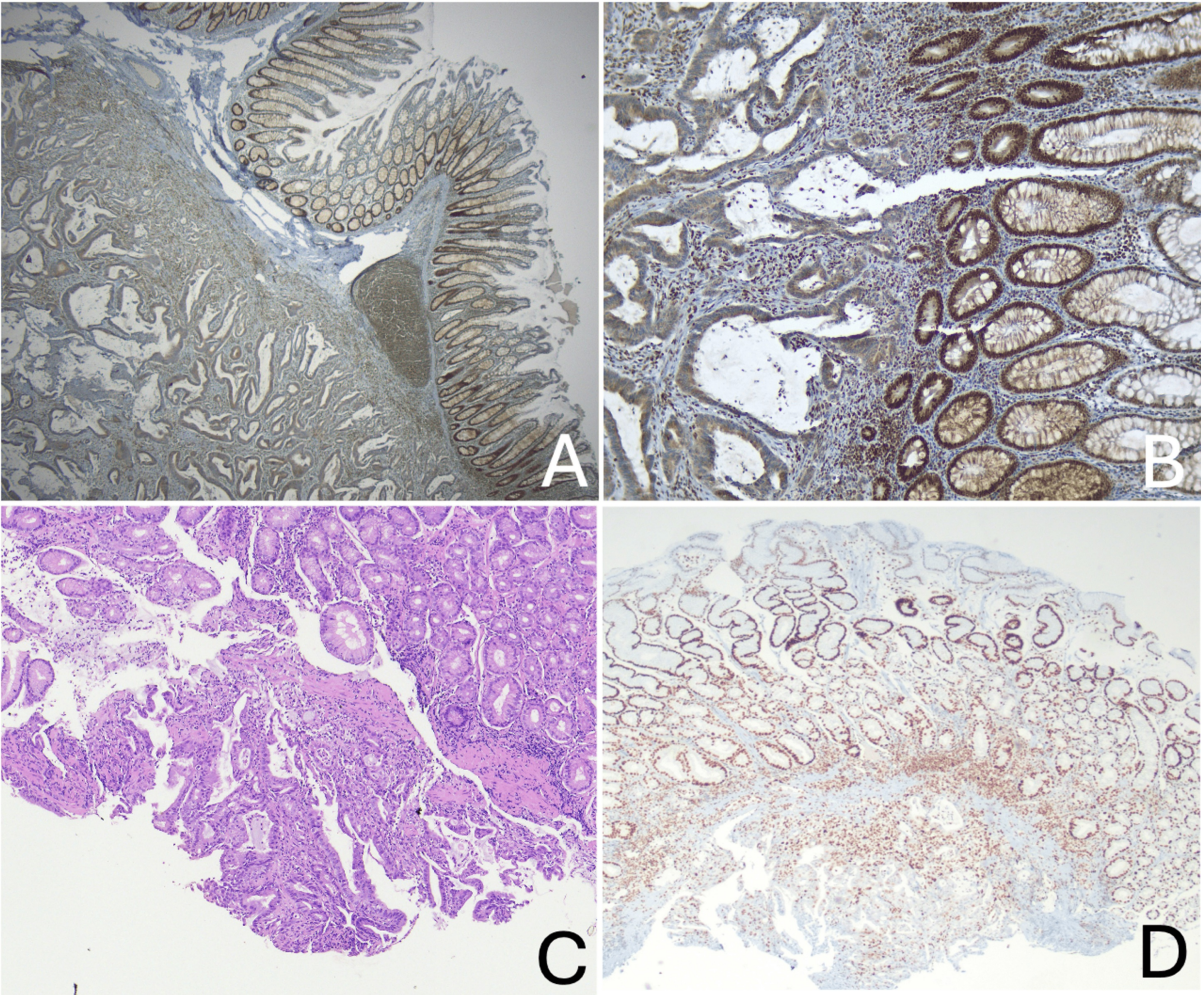
(A and B) IHC examination of the colectomy specimen at age 20, demonstrating loss of MSH2 protein expression on the tumor tissue (A: 2.5x; B: 10x). (C) Η&E showing gastric mucosa (foveolar type) with focal infiltration in deeper areas of the tissue by a malignant epithelial neoplasm with morphological features of a moderately differentiated adenocarcinoma (40x). (D) IHC reveals normal expression of MSH2 protein on the tumor tissue (40x). IHC: immunohistochemistry; H&E: hematoxylin and eosin

The patient underwent a right hemicolectomy, and histopathology of the surgical specimen confirmed the presence of colon adenocarcinoma (stage II: pT3N0, *American Joint Committee on Cancer* (AJCC), eighth edition) [4]. Immunohistochemistry (IHC) revealed the loss of MSH2 protein expression (Figures 1A, 1B).

**Figure 2:**
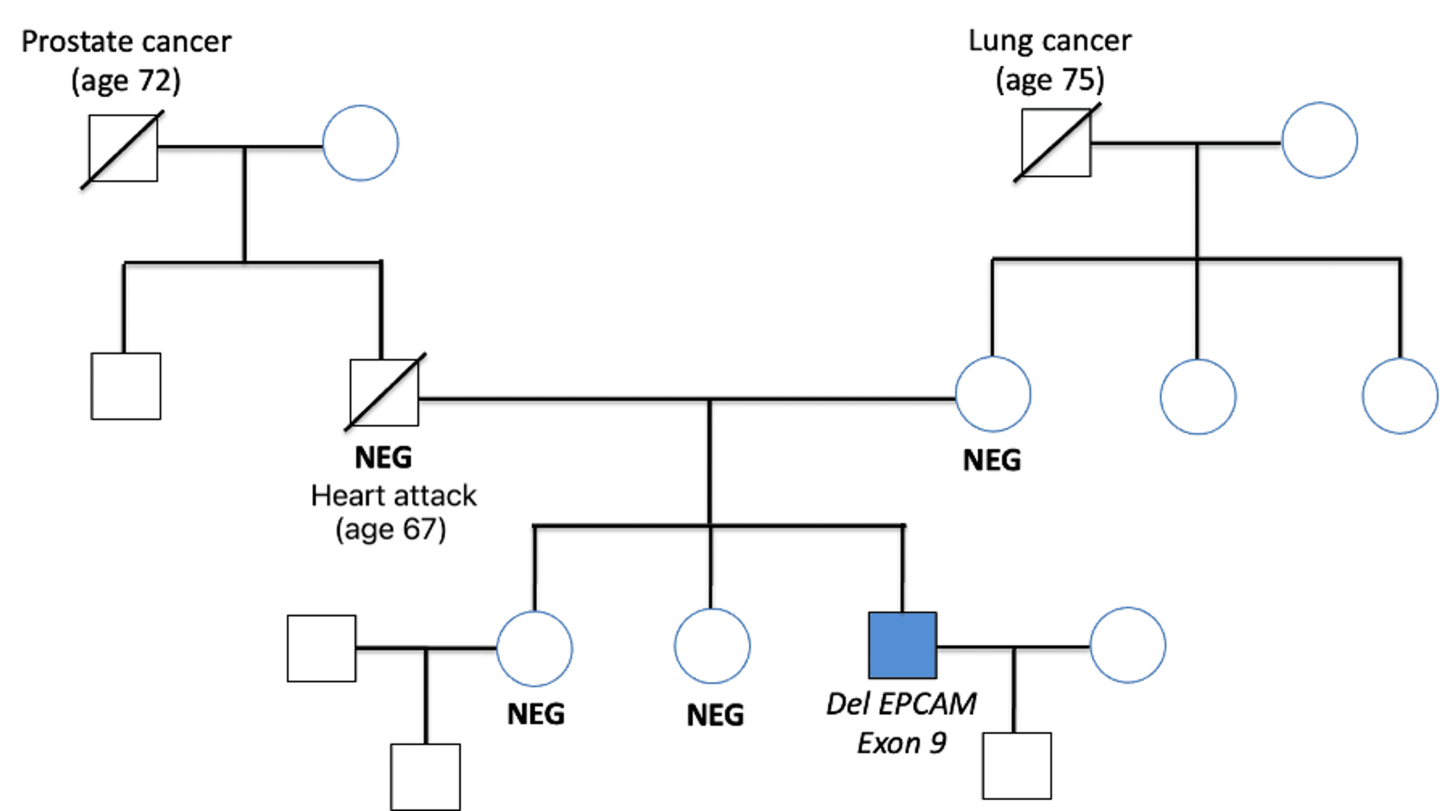
The patient’s family tree. Our patient is highlighted by a blue box. Individuals labeled "NEG" have been tested and found negative for germline Lynch syndrome-associated mutations.

Given the young age at diagnosis, next-generation sequencing (NGS) analysis of the blood sample was performed, revealing a germline deletion of exon 9 of the *EPCAM* gene along with a loss of exon 1 of the *MSH2* gene, consistent with a diagnosis of LS. However, upon contacting the laboratory, the apparent loss of exon 1 of *MSH2* was attributed to a multiplex ligation-dependent probe amplification (MLPA) artifact due to the proximity of the probe to the *EPCAM* 3′ region, and the LS diagnosis was interpreted as resulting from epigenetic silencing of *MSH2* rather than from a structural deletion of the gene. The patient’s family history was negative for malignancies associated with LS, and all first-degree relatives tested negative for LS (including the *EPCAM-MSH2* gene deletion) or constitutional MMR syndrome (CMMRD)-associated mutations (Figure 2). The patient was referred to a Genetics Specialist and was under close monitoring with serial CT scans and GI endoscopies.

**Figure 3:**
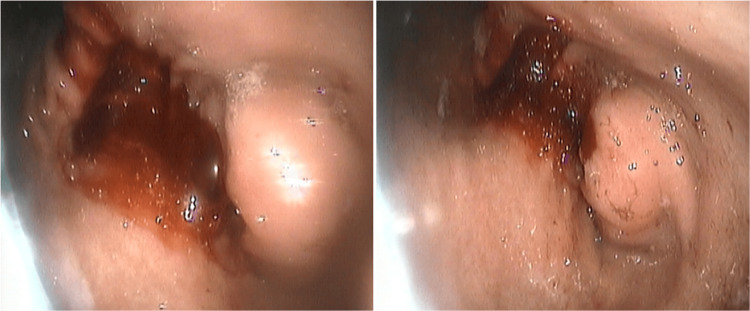
Images obtained at the age of 33 during follow-up endoscopic evaluation demonstrating extrinsic compression of the pylorus wall, suggestive of an external lesion.

At the age of 33 years, during a follow-up upper GI endoscopy, extrinsic pressure to the stomach wall was noted (Figure 3).

This finding prompted an endoscopic ultrasound-guided fine-needle biopsy (EUS-FNB), which revealed a 38x35 mm mass located external to the pylorus. Histopathological examination suggested an adenocarcinoma of unknown primary site (CUP). Immunohistochemical analysis showed positive staining for cytokeratin 7 (CK7) and mucin 1 (MUC1), and negative staining for cytokeratin 20 (CK20), mucin 20 (MUC20), and special AT-rich sequence-binding protein 2 (SATB-2), suggesting the tumor most likely originates from the pancreas or the biliary tract (Figure 1C). Immunohistochemically, no loss of expression in MMR proteins was detected in the tumor tissue (Figure 1D).

The lesion displayed hypermetabolic activity on fluorodeoxyglucose positron emission tomography (FDG PET) scan (SUVmax: 18.2) (Figure 3A), which also revealed multiple lymph node and peritoneal metastases. Tumor markers carcinoembryonic antigen (CEA) and carbohydrate antigen 19-9 (Ca 19-9) were as follows: CEA: 15.22 (reference range: 0-10 ng/mL) and Ca 19-9: 6629 (reference range: 0-37 U/dL).

**Figure 4:**
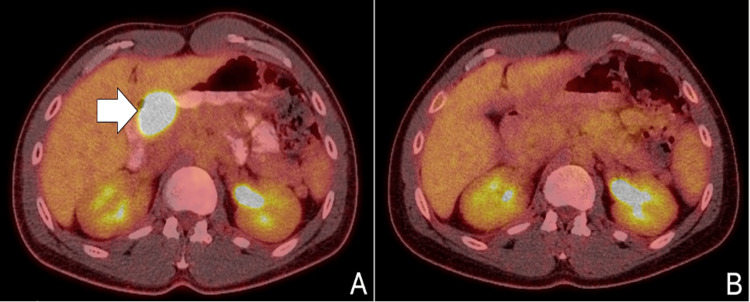
(A) Initial staging FDG PET scan demonstrating a hypermetabolic lesion located externally to the pylorus (white arrow), along with multiple lymph node and peritoneal metastases. (B) Restaging FDG PET scan after 12 cycles of systemic therapy with FOLFIRINOX-durvalumab, demonstrating the absence of hypermetabolic activity in the abdomen, indicative of complete response according to the RESIST 1.1 criteria. FOLFIRINOX: FOLinic acid (Leucovorin), Fluorouracil (5-FU), IRINotecan hydrochloride, and OXaliplatin; FDG PET: fluorodeoxyglucose-positron emission tomography; RESIST 1.1: Response Evaluation Criteria in Solid Tumors 1.1 [5]

The case was discussed in a multidisciplinary tumor board (MDT) meeting, which decided to treat it as a metastatic tumor of unknown primary site. The patient received 12 cycles of FOLFIRINOX (folinic acid, 5-fluouracil, irinotecan, and oxaliplatin), alongside immunotherapy with durvalumab, and restaging FDG PET scan confirmed the absence of hypermetabolic activity (Figure 4), indicative of complete response (CR) according to the Response Evaluation Criteria in Solid Tumors (RESIST) 1.1 criteria [5], whereas tumor markers had returned to normal limits (CEA: 3.02 ng/mL, Ca 19-9: 11.35 U/dL). The patient continues therapy with durvalumab, maintaining biochemical response and radiographic disease control for nearly 14 months since the initiation of treatment. He recently fathered a son who will be offered testing for LS-associated mutations upon reaching adulthood, should he choose to undergo it. Furthermore, tumor NGS was deferred due to cost considerations, but may be pursued in the event of disease recurrence. 

## Discussion

MMR genes *MLH1*, *MSH2*, *MSH6*, and *PMS2* play a crucial role in maintaining genomic stability by correcting DNA replication errors [6]. Inherited mutations in these genes disrupt the DNA repair process, leading to an accumulation of mutations in coding and non-coding regions, a condition known as MSI [7]. MSI is the hallmark of LS, an autosomal dominant disorder that confers susceptibility to various malignancies, including colorectal and endometrial, as well as small intestine, gastric, pancreatobiliary cancers, among others [7,8]. As per the National Comprehensive Cancer Network (NCCN) guidelines, intensive surveillance strategies are currently employed to minimize cancer risk in mutation carriers, including, but not limited to, annual or biennial colonoscopy since the age of 25, annual magnetic resonance imaging (MRI) with magnetic resonance cholangiopancreatography (MRCP), annual endoscopic ultrasound (EUS), and annual skin examination, and esophagogastroduodenoscopy (EGD) every two years [9].

Recently, germline alterations in the *EPCAM* gene have emerged as a novel mechanism of MSI, comprising about 3% of known LS cases [8,10]. The 3’ end of the *EPCAM* gene is located just 15kB upstream of the *MSH2* gene promoter [11]. An *EPCAM* exon 9 deletion removes the transcription termination signal at the 3′ end of the *EPCAM* gene [12]. As a result, RNA polymerase continues transcription beyond *EPCAM* into the neighboring *MSH2* gene, producing an *EPCAM-MSH2* fusion transcript [12]. This transcriptional read-through induces hypermethylation of the *MSH2* gene promoter, leading to epigenetic silencing of the *MSH2* gene [13,14]. A study by Kuiper et al. demonstrated that the majority of 3’ deletions occur on the exon 8 or 9 of the *EPCAM* gene [11].

Regarding cancer risk in *EPCAM* deletion carriers, cumulative CRC risk is equivalent to that in *MSH2* mutation carriers, calculated to 75% until the age of 70, whereas the endometrial cancer risk is up to 12%, which is lower compared to that of MSH2 mutation carriers [15,16]. In terms of extracolonic tumors, *EPCAM* deletion carriers face a higher risk for gastric (18%), small bowel (5%), pancreatic (4%), ovarian (15%), and urinary tract cancer (20%) [17]. The lifetime risk for bile duct cancer, which is 2% in LS, is not well-documented for *EPCAM* deletion carriers [16].

The diagnosis of LS is based on several methods, including IHC, polymerase chain reaction (PCR)-based assays, and molecular testing [17]. IHC detects the lack of staining for any of the MMR proteins using specific monoclonal antibodies against MLH1, MSH2, MSH6, or PMS2. Another diagnostic hallmark of LS is the presence of MSI, which is identified by comparing the length of microsatellites in tumor tissue to those in normal tissue using specific PCR-based assays. Furthermore, molecular testing through NGS can detect germline gene alterations associated with LS [10].

There are many criteria that were established to identify families that may benefit from genetic testing and surveillance for LS-associated cancers. According to the Revised Bethesda Guidelines, CRC patients are elligilbe for MSI testing if any of the following criteria is met: (i) individuals younger than 50 years old, (ii) presence of synchronous or metachronous colorectal or other LS-associated tumors regardless of age, (iii) CRC with high-MSI histology diagnosed in patients younger than 60 years old, (iv) individuals with one or more first-degree relatives with LS-associated tumors, with at least one of the cancers being diagnosed before the age of 50 years, and (v) two or more first or second-degree relatives with LS-associated tumors, irrespective of age [7]. The Amsterdam Criteria for diagnosing LS contain criteria for other extracolonic LS-associated tumors and include the following: (i) At least three relatives with histologically verified LS-associated cancers (such as colorectal, endometrial, stomach, ovarian, ureter/renal pelvis, brain, small bowel, hepatobiliary tract, and sebaceous skin tumors), with one being a first-degree relative of the other two. (ii) These cancers should span at least two successive generations, and (iii) At least one of the HNPCC-associated cancers should be diagnosed before the age of 50 [10]. Patients satisfying all three criteria are diagnosed with LS. Our patient satisfied one of the Bethesda criteria for MSI testing (age <50 years old) but did not meet the Amsterdam criteria for LS diagnosis. However, he was diagnosed with an exon 9 deletion mutation on the *EPCAM* gene, a gene implicated in LS, and his CRC was characterized as MSI-high.

To date, the surveillance strategy for CRC in *EPCAM* gene deletion is similar to that for other LS carriers [18]. However, due to the significantly lower incidence of endometrial cancer in *EPCAM* mutation carriers, surveillance or risk-reducing surgery for endometrial cancer is not recommended [18]. Data on surveillance of other tumors, including gastric, pancreatobiliary, and urothelial cancers, with abdominal ultrasound, EGD, or urine cytology remain scarce, and decisions should be based on a personalized approach [18]. Genetic counseling is strongly recommended in all cases, in order to identify mutation carriers in the patient’s family.

Historically, MSI-high tumors are associated with a lack of response to chemotherapy [9]. However, an excellent response to immune checkpoint inhibitors is linked to the overproduction of neoantigens that are recognized by the cytotoxic T-lymphocytes and generate an immune reaction [3,17,19]. This has led to the approval of pembrolizumab for MSI-high or MMR-deficient solid tumors after progression on conventional therapy [9]. To date, there is insufficient data to determine specific prognosis and response to therapy in *EPCAM* gene mutation carriers. Our case demonstrated an excellent response to chemo-immunotherapy, with a response duration of more than 12 months.

Our patient was found to have a germline *EPCAM* exon 9 deletion, which appears to be a de novo mutation. He was diagnosed with two LS-associated primary tumors, one of which demonstrated a loss of MSH2 protein on IHC. The second tumor was also considered potentially LS-associated given the patient’s confirmed germline status, the young age at diagnosis, the exceptional response to therapy, and the possibility of mosaic or heterogeneous MSH2 expression within the tumor, reflecting incomplete or tissue-specific promoter methylation [6]. Our patient received a personalized chemo-immunotherapy regimen tailored to the most probable site of origin of his carcinoma and the diagnosis of MSH2 protein loss. To our knowledge, this is the first reported case worldwide where such a regimen has been employed, resulting in a complete response, highlighting the potential for individualized treatment strategies in achieving successful outcomes in rare genetic conditions.

## Conclusions

The case underscores the genetic heterogeneity of LS and illustrates that *EPCAM* deletions represent an additional mechanism leading to MMR deficiency. Comprehensive genetic testing in potential carriers, including *EPCAM* analysis, is crucial for accurate diagnosis and tailored management. The observed complete response to a chemo-immunotherapy regimen further highlights the potential of personalized therapeutic strategies, especially in tumors with unique molecular signatures. Expanding the current LS diagnostic criteria to incorporate rare genetic mechanisms such as *EPCAM* deletions is essential, and the role of reference centers and molecular tumor boards is pivotal in providing expert guidance for accurate diagnosis and precision treatment planning.
